# Vector competence of *Aedes aegypti* for different strains of Zika virus in Argentina

**DOI:** 10.1371/journal.pntd.0007433

**Published:** 2019-06-12

**Authors:** Melisa Berenice Bonica, Silvina Goenaga, María Laura Martin, Mariel Feroci, Victoria Luppo, Evangelina Muttis, Cintia Fabbri, María Alejandra Morales, Delia Enria, María Victoria Micieli, Silvana Levis

**Affiliations:** 1 Centro de Estudios Parasitológicos y de Vectores (CEPAVE-CONICET), Universidad Nacional de La Plata, La Plata, Buenos Aires, Argentina; 2 Instituto Nacional de Enfermedades Virales Humanas “Dr. Julio Maiztegui” (INEVH-ANLIS), Pergamino, Buenos Aires, Argentina; Fundaçao Oswaldo Cruz, BRAZIL

## Abstract

The importance of Zika virus (ZIKV) has increased noticeably since the outbreak in the Americas in 2015, when the illness was associated with congenital disorders. Although there is evidence of sexual transmission of the virus, the mosquito *Aedes aegypti* is believed to be the main vector for transmission to humans. This species of mosquito has not only been found naturally infected with ZIKV, but also has been the subject of study in many vector competence assays that employ different strains of ZIKV around the world. In Argentina, the first case was reported in February 2016 and a total of 278 autochthonous cases have since been confirmed, however, ZIKV virus has not been isolated from any mosquito species yet in Argentina. In order to elucidate if Argentinian *Ae*. *aegypti* populations could be a possible vector of ZIKV, we conducted vector competence studies that involved a local strain of ZIKV from Chaco province, and a Venezuelan strain obtained from an imported case. For this purpose, *Ae*. *aegypti* adults from the temperate area of Argentina (Buenos Aires province) were fed with infected blood. Body, legs and saliva were harvested and tested by plaque titration on plates of Vero cells for ZIKV at 7, 11 and 14 days post infection (DPI) in order to calculate infection, transmission, and dissemination rates, respectively. Both strains were able to infect mosquitoes at all DPIs, whereas dissemination and transmission were observed at all DPIs for the Argentinian strain but only at 14 DPI for the Venezuelan strain. This study proves the ability of *Ae*. *aegypti* mosquitoes from Argentina to become infected with two different strains of ZIKV, both belonging to the Asian lineage, and that the virus can disseminate to the legs and salivary glands.

## Author summary

Zika virus is a flavivirus transmitted by mosquitoes, isolated for the first time in the Ziika Forest in Uganda in 1947 from a rhesus macaque monkey. The disease is usually asymptomatic, but sometimes it causes a mild illness that comes with fever, rash, joint pain, and conjunctivitis. The World Health Organization focused the attention on this virus after the outbreak in the Americas, when the virus was linked to microcephaly and serious neurological diseases, including Guillain-Barré syndrome. *Aedes aegypti* was incriminated as the main vector of the virus as it was found both naturally and experimentally infected. This mosquito species was declared eradicated in Argentina by 1970 but re-emerged in 1989. Recent studies found a peculiarity in the genetics of Argentinian *Ae*. *aegypti* populations that consists in a combination between both subspecies: *Ae*. *aegypti formosus* and *Ae*. *aegypti aegypti*. Our study tries to elucidate if *Ae*. *aegypti* from Argentina are able to transmit the virus in order to add these mosquitoes to the list of possible vectors of ZIKV and, in future prospect, orient to fight the virus by controlling the vector.

## Introduction

Zika virus (ZIKV) is a single-stranded positive sense RNA virus that was first isolated in 1947 in the Ziika Forest in Uganda from a sentinel Rhesus monkey [1,2]. One year later, this arthropod-borne virus (arbovirus) member of the genus *Flavivirus* was isolated from *Aedes africanus* mosquitoes in the same forest, suggesting the mosquito as vector of the virus [2]. Outside Africa, ZIKV was isolated for the first time from *Aedes aegypti* in Malaysia in 1966, providing evidence of transmission by an urban vector [3]. Since then, human cases were reported occasionally in Africa and Asia, until 2007, when a massive outbreak was reported in Yap Island where the virus seems to have emerged from its sylvatic cycle to a rural habitat, causing fever, rash, conjunctivitis, and arthralgia [4,5]. Successively, a ZIKV outbreak that affected approximately 11% of the population occurred in French Polynesia in 2013. During this outbreak, the Guillain-Barré syndrome (GBS) was associated with ZIKV for the first time [5].

In the Americas, the virus was introduced in Brazil [6], probably after the World Cup soccer games held between June and July 2014, or during the 2014 World Sprint Championship held in Rio de Janeiro in August, in which four Pacific countries participated [7]. The first cases in patients were reported in 2015 [8]. Since this outbreak, the virus had spread all over the country by the end of 2015, reaching other 28 countries in South and Central America by February 2016 [5]. The interest in ZIKV has increased when the infection was correlated with severe congenital disorders such as microcephaly (MC) and other neurological malformations in fetuses and newborns [9], especially when mothers are infected during the first trimester of pregnancy [10]. Due to the consequences caused by MC that severely affects cognitive and motor skills, many families are force to leave their jobs to care for their children having an impact in their socioeconomic status [5].

Although there is evidence that sexual intercourse is a route of transmission between humans [11,12], the mosquito bite is still believed to be responsible for the dispersion of the virus, with humans as amplification hosts in endemic and epidemic zones during the urban cycle [13]. Sixteen different *Aedes spp*. mosquitoes were found naturally infected in the field with ZIKV. Among all, *Ae*. *aegypti* is considered to be the predominant species in the transmission of the virus, probably because it is close associated with humans in urban areas [14]. Additionally, ZIKV has been isolated from *Ae*. *aegypti* in field-caught specimens, although very occasionally, with evidence of vertical transmission detected in a few cases [3,15–20]. Furthermore, *Ae*. *aegypti* was confirmed to be competent in the transmission of ZIKV in a large number of experimental assays [4,14], with differences in transmission efficiency been attributed to the genetic background of the vector population and the virus strain utilized [4,14,21–24].

**Fig 1:**
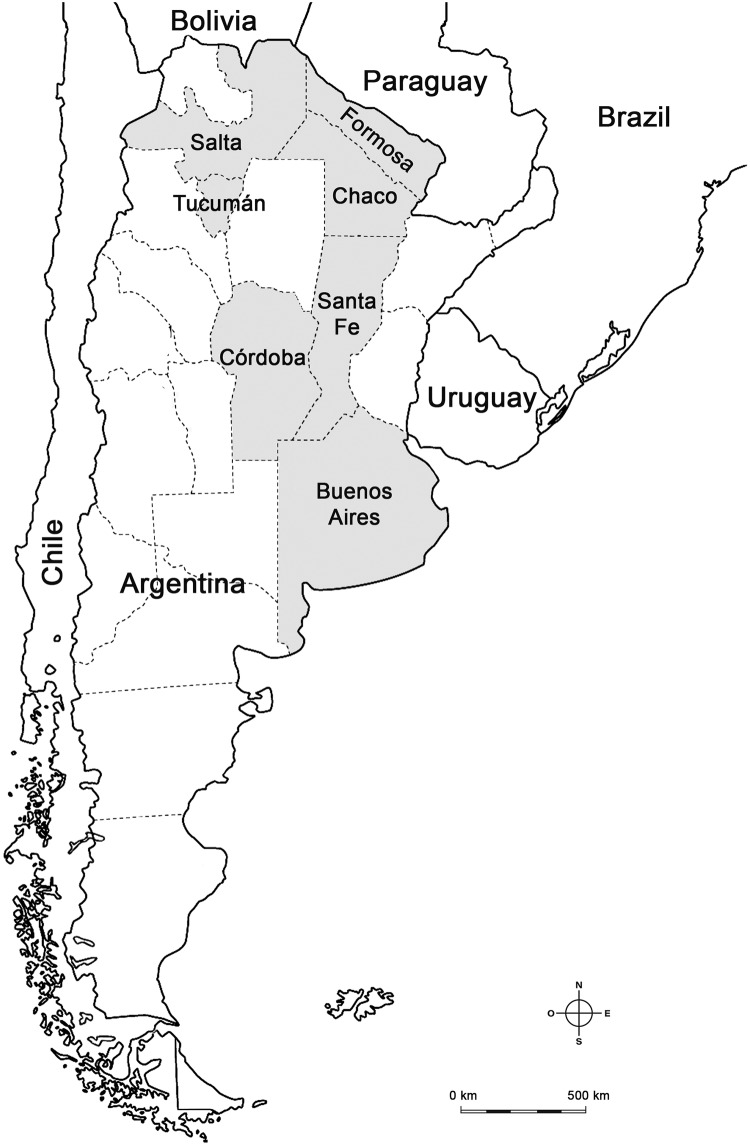
Distribution of autochthonous cases of ZIKV in Argentina. The figure shows Argentinian provinces with autochthonous cases of ZIKV (grey) registered between 2016 and 2018, and bordering countries.

In Argentina, ZIKV was first detected in the Córdoba province (temperate central area) in February 2016, with the case being attributed to the sexual transmission of the virus (Fig 1). A few weeks later, the first outbreak occurred in Tucumán province resulting in 25 confirmed autochthonous cases. During October 2016, the first case of congenital syndrome caused by ZIKV infection was confirmed in a newborn in Tucumán [25]. During the first semester of 2017, 251 autochthonous cases of Zika were registered in Formosa, Salta and Chaco, all provinces located in North Argentina close to the Bolivian and Paraguayan borders (Fig 1) [25]. Additionally, a MC case was detected in a newborn in Santa Fe province. Although the mother had no recent travel records, additional studies confirmed her ZIKV infection status [25]. Interestingly, during 2018 ZIKV circulation seems to be confined to Salta province where 54 locally acquired cases where confirmed. Additionally, in Buenos Aires province the only ZIKV case without travel antecedent was registered until now [26].

Due to the lack of entomological surveillance studies in Argentina during the ZIKV outbreak, as well as no vector competence studies in Argentinian mosquitoes, we aim to evaluate the potential role of *Ae*. *aegypti* in ZIKV transmission. To do so, we challenged a population of *Ae*. *aegypti* from La Plata in the Buenos Aires province against two different strains of ZIKV, in order to determine not only if the mosquito population from this region would be capable of transmitting the virus, but also to test for different levels of vector competence using distinct viral strains. One strain derives from an imported case from Venezuela, whereas the other strain was isolated from an autochthon case from Chaco province in Argentina.

## Methods

### Mosquitoes and viral strains

*Aedes aegypti* mosquitoes employed in this study are derived from a laboratory colony established from La Plata in Buenos Aires province (Argentina). The colony was originated in 2014 from larvae mosquitoes originally collected from La Plata cemetery, and since then it has been maintained at Centro de Estudios Parasitológicos y de Vectores (CEPAVE) in La Plata. Periodical introgressions of field mosquitoes from the same area of the city are incorporated seasonally to the colony in order to keep the genetic background as close as possible to the field. Multilocus genotype analysis was carried out with some individuals from the same area of collection [27]. For this study we selected two Zika virus strains: one strain (ZIKV-VEN) was isolated from a patient who travelled from Venezuela to Argentina (strain ARCB116141, GenBank accession no. MK637519), whereas the other strain (ZIKV-ARG) was isolated from a patient in Chaco where the virus circulated among the population (strain ARCH125797, GenBank accession no. MK637518). These strains show a nucleotide identity of 99.5% and an amino acid identity of 100% for a fragment that enclosed the last part of the capsid (C), the precursor membrane segment (prM), and the first part of the envelope (E).

The viral stock was prepared after four passages on Vero cells and frozen at −86°C before being employed in oral infections. The titers of each strain were analyzed by plaque assays on 12-well plates of Vero cells. The titer for the ZIKV-VEN strain was 7.17 log_10_ PFU/ml whereas the titer for the ZIKV-ARG was 5.3 log_10_ PFU/ml. These values are close to the range of viremia that has been previously reported for ZIKV infection [28].

### Oral infection

Adult mosquitoes were maintained via incubation at 27 ± 1°C, 70 ± 10% RH, 16:8 hours light:dark cycle, and supply with sugar and water, except for a period of 24 hours of starvation prior to the oral infection. Four-five days after emergence, mosquitoes were offered a blood meal supplied by a glass artificial feeder which allows maintaining the blood temperature at 37°C. Blood meals were comprised of 4 ml of ovine blood (Laboratorio Alfredo Gutierrez, C.A.B.A., Argentina), 0.5 ml of sucrose 50%, and 0.5 ml of previously frozen cell culture supernatant containing ZIKV. Mosquitoes were fed for one hour. Engorged females were counted and separated in three cardboard cages to be analysed at 7, 11, and 14 days post infection (DPI). The cages were placed back into the incubator at the same conditions of temperature and humidity until sample collection at each time point. Each group was offered sugar and water *ad libitum*. All the infections assays were performed in biosafety level 3 facilities at Instituto Nacional de Enfermedades Virales Humanas (INEVH) in Pergamino.

### Mosquito samples processing

At 7, 11 and 14 DPI mosquitoes were anesthetized with triethylamine as previously described [29], and bodies, legs, and saliva were harvested from each mosquito. Different time points were selected in order to determine the extrinsic incubation period (EIP), which corresponds to the time between oral infection and presence of virus in saliva. The proboscis of each immobilized mosquito was inserted into a capillary tube containing 5 μl of Minimum Essential Medium (MEM) supplemented with 20% of fetal bovine serum (FBS). After 30 min of salivation, the proboscis was removed from the capillary tubes, and legs and bodies separated into individual tubes. Each capillary tube containing salivary expectorate was collected from the capillary into a tube containing 300 μl of MEM supplemented with 20% FBS. All samples were stored at -86 °C until processing. Bodies and legs were each homogenized separately in microcentrifuge tubes containing 1.4 mm ceramic beads and 1 ml MEM with 20% FBS, for one min at 20 cycles per second using a Bead Ruptor 24 Elite (OMNI international, Kennesaw, Georgia, USA). Homogenates were clarified by centrifugation at 5000xG for 10 min at 4 °C. In order to detect ZIKV infectious virions, all samples were analysed by plaque titration on 12-well plates of Vero C76 cells. Titration was performed as previously described [30]. Briefly, tenfold serial dilutions of each sample in MEM supplemented with 2% FBS and antibiotics were added in a confluent Vero C76 monolayers attached to 12-well plates and incubated for 1 hour with periodic gentle rocking to facilitate virus adsorption at 37°C. The volume of the inoculums was 100 ul in each well. Plaques were incubated undisturbed for 5 days at 37°C. Vital dye neutral red was used at 2% for plaque visualization. The mosquito body was examined to estimate the infection rate (IR), the legs to estimate the dissemination rate (DR), and saliva for the transmission rate (TR) of the virus. IR is defined as the percentage of mosquitoes with infected body among total engorged mosquitoes. DR corresponds to the percentage of mosquitoes that contained infectious virus in their legs among the previously infected mosquitoes detected. TR is reported as the percentage of mosquitoes that contained infectious virus in the saliva, among mosquitoes with disseminated infection. Transmission efficiency (TE) refers to the proportion of mosquitoes with infectious saliva among the total number of engorged mosquitoes. Differences in the IR, DR and TR between the two strains (ZIKV-VEN and ZIKV-ARG) were compared by a Fisher exact test, considering statistically significant p-value < 0.05. Comparisons of viral titers in body and legs between both strains were performed at 14 DPI by using Student’s t-test or permutation test according to data normality. All analyses were performed using R software (v. 3.5.0) [31–34].

## Results

The total number of mosquitoes employed in the assay for both strains were similar (60 and 61 specimens) (Table 1). Infection was successful for both ZIKV strains in all three DPIs, and IR varied from 15.8% to 50% for ZIKV-ARG and from 11.1% to 61.8% for the ZIKV-VEN strain (Table 1). Virus dissemination was found at 7, 11 and 14 DPI for the ZIKV-ARG strain, with a total of 14 mosquitoes confirmed to have disseminated virus during the experiment (60.9%). On the other hand, for the ZIKV-VEN strain, dissemination was observed only at 14 DPI, where virus dissemination was detected in 52.4% of the mosquitoes. Although the proportion of ZIKV-infected saliva from *Ae*. *aegypti* was low for both strains, transmission was still observed. One of 11 samples was found positive for ZIKV-VEN strain in saliva at 14 DPI, whereas one sample of 6 was found positive for ZIKV-ARG in saliva by the same DPI. However, unlike the imported strain, TR was detected at all DPIs for the ZIKV-ARG strain. The ZIKV-ARG strain exhibited a minimum extrinsic incubation period (EIP) of 7 days, while the EIP for ZIKV-VEN was 14 days. Finally, the total TE was 6.6% for ZIKV-ARG and 1.7% for ZIKV-VEN. No significant differences were detected for the total IR (p-value = 0.71), DR (p-value = 0.61), TR (p-value = 0.34) and TE (p-value = 0.71) between both strains. Virus titer in saliva was detected by plaque assay in all samples despite the results of IR and DR. We did not find any saliva sample positive where infection and dissemination were negative.

**Table 1:** IR, DR, TR, and TE of ZIKV-ARG and ZIKV-VEN at 7, 11 and 14 DPIs in *Ae*. *aegypti* from La Plata, Argentina. No statistical differences were found between both strains.

Strain	DPI	N° Fed mosq.	Infected mosq.	IR (%)	Disseminated mosq.	DR (%)	Transmitting mosq.	TR (%)	TE (%)
ZIKV-ARG	7	19	3	15.8	1	33.3	1	100.0	5.25
	11	20	10	50.0	7	70.0	2	28.6	10.0
	14	22	10	45.5	6	60.0	1	16.7	4.5
	Total	61	23	37.7	14	60.9	4	28.6	6.6
ZIKV-VEN	7	18	2	11.1	0	0.0	0	0	0
	11	8	2	25.0	0	0.0	0	0	0
	14	34	21	61.8	11	52.4	1	9.1	2.9
	Total	60	25	41.7	11	44.0	1	9.1	1.7

For both ZIKV strains the mean titers in body, legs and saliva were calculated for each DPI (Table 2). Furthermore, comparisons between two strains were performed at 14 DPI for body and legs, when data was available. There were not significant differences in the body between both strains (Student’s t-test, t = -0.47, df = 26, p-value = 0.64). When comparing viral titers between legs, a permutation test was preferred due to the low number of samples. In this case, significant differences were found between both strains, being the viral titers in legs for ZIKV-VEN higher than those for ZIKV-ARG (Z = -2.04, p-value = 0.04). Additionally, comparisons between legs and saliva showed that the average viral titers in saliva dropped respect to the legs 8.4% for ZIKV-ARG, and 40.7% for ZIKV-VEN.

**Table 2:** Mean titers in body, legs and saliva in mosquitoes for ZIKV strains from Argentina and Venezuela at each DPI (7, 11 and 14).

Strain	DPI	Mean body titer (log_10_ PFU/ml) ± SD (N)	Mean legs titer (log_10_ PFU/ml) ± SD (N)	Mean saliva titer (log_10_ PFU/ml) ± SD (N)
ZIKV-ARG	7	3.8 ± 0.0 (3)	1.3 (1)	1 (1)
	11	3.5 ± 1.5 (9)	1.2 ± 0.2 (7)	1.4 ± 0.1 (2)
	14	4.1 ± 1.2 (10)^a^	1.7 ± 0.8 (6)^b^	1.3 (1)
	Total	3.8 ± 1.2 (22)	1.4 ± 0.6 (14)	1.3 ± 0.2 (4)
ZIKV-VEN	7	3.4 ± 0.6 (2)	NA (0)	NA (0)
	11	5.3 ± 0.1 (2)	NA (0)	NA (0)
	14	4.3 ± 0.8 (18)^a^	2.5 ± 0.6 (11)^c^	1.5 (1)
	Total	4.3 ± 0.9 (22)	2.5 ± 0.6 (11)	1.5 (1)

NA: not applicable.

^a^ no statistical differences were found (Student’s t-test).

^b,c^ statistical differences were found (Permutation test).

## Discussion

The ZIKV outbreak in Brazil in 2015 triggered an international alarm, especially when neurological disorders and microcephaly in newborns were associated with the infection [5]. Due to its proximity to Brazil, and the presence of the implicated vector, Argentina also focused the attention on this neglected disease. Interestingly, 137,288 Zika autochthonous cases were reported by the Brazilian Ministry of Health by January 2018, whereas in Argentina 278 autochthonous cases were confirmed by the Argentinian Ministry of Health in the same period, confined to five provinces in the Northern region of the country [35]. In America, *Ae*. *albopictus* was found naturally infected with ZIKV in Brazil, while *Ae*. *aegypti*-infected mosquitoes were detected in Brazil, Ecuador and Mexico [17,20,36,37]. Vector competence studies that involved *Ae*. *aegypti* population from different countries corroborated the efficiency of this species in transmitting different strains of ZIKV, however competence varies greatly, and depending mainly on mosquito origin, Zika strain and type of blood meal used [21–24,38–41]. Vector competence of *Ae*. *aegypti* for ZIKV has been evaluated through all five continents. In Africa, mosquitoes populations from Senegal and Nigeria were tested for 14 different ZIKV strains, all of them infected this species but only two strains reached the saliva. In Asia, mosquitos from Singapore were able to transmit three strains of ZIKV showing an EIP of 3 and 4 days. Three ZIKV strains were also transmitted by *Ae*. *aegypti* from Australia and French Polynesia. In Europe a population of *Ae*. *aegypti* from Madeira Island was tested against two different strains of ZIKV; both strains infected the mosquitoes but only one of them was transmitted (EIP = 9). Finally, vector competence studies were also carried out in Mexican and Brazilian mosquitoes, which were able to transmit seven and three different strains of ZIKV, respectively [13]. These studies, together with the isolation of ZIKV from field-collected mosquitoes, confirm *Ae*. *aegypti* as the main vector of ZIKV [13,14]. In Argentina, ZIKV has not yet been isolated in the field from any mosquito species, and no vector competence studies were performed so far with local ZIKV strains.

In this study we evaluated the vector competence of a local population of *Ae*. *aegypti* from the temperate area of Argentina (La Plata), for two different Zika virus strains both belonging to the Asian lineage. One strain was isolated from a patient who has travelled to Venezuela (ZIKV-VEN), whereas the other strain was isolated in Chaco, Argentina, during the outbreak in 2017 (ZIKV-ARG). We demonstrated that the Argentinian *Ae*. *aegypti* population is able to be infected by both ZIKV-VEN and ZIKV-ARG strains. Despite the fact that the proportion of mosquitoes infected with these strains was relatively high, the TR remains very low for both strains. Moreover, the titers in the transmitting mosquitoes were also very low. However, it should be considered that due to the low number of infected mosquitoes at 7 DPI for both strains, and 11 DPI for ZIKV-VEN, DR and TR at these time points may not reflect the actual susceptibility of the population used in this study. For the Argentinian strain we detected an overall TE (6.6%) that was slightly higher than the overall TE for the Venezuelan strain (1.7%). These data are closer to the TE detected in *Ae*. *aegypti* from Rio de Janeiro (10%), than the TE found for ZIKV strains transmitted by *Ae*. *aegypti* populations from Los Angeles (53–75%) [22,42]. One possible explanation for the low levels of transmission could be due to the employment of frozen virus stocks. The difficulty in conducting the experiment with fresh virus leaves us questions about whether the susceptibility of the strains may vary according to a different response to the virus freeze/thaw, leading to a decrease of transmission efficiency [23,43,44]. Another factor that could influence on the transmission is the genetics of the mosquito. In fact, *Ae*. *aegypti s*.*l*. is divided into two genetic units which correspond to the standard defined subspecies: *Ae*. *aegypti formosus*, in Africa, and *Ae*. *aegypti aegypti* outside Africa. In Argentina, a mixture between both subspecies was detected in four different populations, including that from La Plata [45]. The fact that Argentinian populations have an African background could be an explanation for the low transmission of ZIKV, since some *Ae*. *aegypti* populations from Senegal were not competent for the transmission of this virus [46]. The circulation of the virus in Argentina was very low compared to Brazil. Interestingly, during 2016, Argentina had the greatest Dengue outbreak in magnitude and geographical dissemination to date. The infection spread through 15 provinces of the center and north of the country, with 41,207 confirmed cases. This situation could have affected the detection of ZIKV infection from both the clinical aspect and the serological studies (IgM cross reactivity) [47]. In Argentina, laboratory assays indicates high vector competence for DENV-2 of *Ae*. *aegypti* populations from both subtropical and temperate areas. Mosquitoes from the subtropics were even more efficient that those of temperate Argentina [48]. Additionally, vector competence studies for DENV and chikungunya virus (CHIKV) in genetically distinct populations of *Ae*. *aegypti* from Argentina (one of these belong to the same population that we used in this study), showed a large variability in vector competence for these viruses. In particular, La Plata *Ae*. *aegypti* were highly refractory to CHIKV infection and even at mean temperatures (higher than the specific-site temperature for La Plata) the population was more refractory than other populations for both pathogens [27].

It is remarkable that although Buenos Aires is the most populated province in the country, and many imported cases were diagnosed from travellers who arrived to the province from all over the world, and considering that the abundance of *Ae*. *aegypti* has increased during the last 20 years, only one autochthonous ZIKV case was reported by the Ministry of Health [26,49,50]. Additionally, *Ae*. *aegypti* is the only invasive species in the province since *Ae*. *albopictus* is present only in the Misiones province bordering Brazil [51,52]. The low TE of the La Plata *Ae*. *aegypti* population observed here might be an explanation for the absence of an outbreak in the region.

The various barriers encountered during the extrinsic incubation period could probably influence the infection and replication in different tissues [53]. Our results show that the decrease in the titers in saliva related to those in legs is four times higher in the Venezuelan strain than in the Argentinian, although mean viral titers were significantly higher in legs for ZIKV-VEN than ZIKV-ARG, suggesting differences in the viral strain fitness. Another difference in the fitness between both viral strains is observed by the shorter EIP of the strain that circulated in the country (ZIKV-ARG) compared to the imported strain.

As was mentioned before, ZIKV outbreak in Argentina was simultaneous with DENV outbreak. Further studies will be necessary in order to evaluate the impact of arboviruses coinfections on the epidemiology of these diseases. Moreover, we will explore in future studies the transmission of the virus among other populations of *Ae*. *aegypti* belonging to areas of Argentina where Zika outbreaks were reported. Because of the genetic diversity observed among *Ae*. *aegypti* in Argentina, these populations could be more susceptible to ZIKV transmission than those from La Plata [27]. Furthermore, albeit the attention is focused on *Ae*. *aegypti*, we do not preclude that other species of *Aedes* could be involved in the transmission of the virus, especially in other provinces affected by the outbreak with a higher diversity of *Aedes spp*. mosquitoes than Buenos Aires [54]. Another factor that should be considered is that RNA viruses replicate with low genetic fidelity that results in high mutation rates. If these genetic changes are able to generate new variants, there could be variability in epidemiological fitness and therefore an increment of vector competence [55,56]. Evidence of the importance of mutation in arbovirus is provided by Chikungunya virus, which adapted to the vector *Ae*. *albopictus* after a single adaptive mutation [57], and for West Nile virus (WNV) which with a single-amino acid substitution became resistant to lycorine [58]. Also, the spread and continuous evolution of WNV led to the change from attenuated to virulent phenotype for lineage 2 [59]. For these reasons we could expect ZIKV to adapt further to local *Ae*. *aegypti* populations. Therefore active surveillance on circulating strains and other vector competence studies could contribute to elucidate the dynamics of the virus in this region.

## References

[1] Kuno G, Chang GJJ. Full-length sequencing and genomic characterization of Bagaza, Kedougou, and Zika viruses. Arch Virol. 2007;152: 687–696. 10.1007/s00705-006-0903-z 17195954

[2] Dick GWA, Kitchen SF, Haddow AJ. Zika virus (I). Isolations and serological specificity. Trans R Soc Trop Med Hyg. 1952;46: 509–520. 10.1016/0035-9203(52)90042-4 12995440

[3] Marchette NJ, Garcia R, Rudnick A. Isolation of Zika Virus from *Aedes Aegypti* Mosquitoes in Malaysia. Am J Trop Med Hyg. 1969;12: 411–15. 10.4269/ajtmh.1969.18.411 4976739

[4] Kauffman EB, Kramer LD. Zika virus mosquito vectors: competence, biology, and vector control. J Infect Dis. 2017;216: S976–S990. 10.1093/infdis/jix405 29267910 PMC5853459

[5] Weaver SC, Costa F, Garcia-blanco MA, Ko AI, Ribeiro GS, Saade G, et al Zika virus: history, emergence, biology, and prospects for control. Antivir Res. 2016;130: 69–80. 10.1016/j.antiviral.2016.03.010 26996139 PMC4851879

[6] Campos G, Bandeira A, Sardi S. Zika Virus Outbreak, Bahia, Brazil. Emerg Infect Dis. 2015;21: 1885–1886. 10.3201/eid2110.150847 26401719 PMC4593454

[7] Musso D. Zika virus transmission from French Polynesia to Brazil. Emerg Infect Dis. 2015;21: 1887. 10.3201/eid2110.151125 PMC4593458 26403318

[8] Zanluca C, Melo VC, Mosimann AL, Dos Santos GI, Dos Santos CND, Luz K. First report of autochthonous transmission of Zika virus in Brazil. Mem Inst Oswaldo Cruz. 2015;110: 569–572. 10.1590/0074-02760150192 26061233 PMC4501423

[9] De Carvalho NS, De Carvalho BF, Fugaça CA, Dóris B, Biscaia ES. Zika virus infection during pregnancy and microcephaly occurrence: a review of literature and Brazilian data. Brazilian J Infect Dis. 2016;20: 282–289. 10.1016/j.bjid.2016.02.006 27102780 PMC9425494

[10] Johansson MA, Mier-y-Teran-Romero L, Reefhuis J, Gilboa SM, Hills SL. Zika and the risk of microcephaly. N Engl J Med. 2016;375: 1–4. 10.1056/NEJMp1605367 27222919 PMC4945401

[11] Musso D, Roche C, Robin E, Nhan T, Teissier A, Cao-Lormeau V-M. Potential sexual transmission of Zika virus. Emerg Infect Dis. 2015;21: 359–361. 10.3201/eid2102.141363 25625872 PMC4313657

[12] McCarthy M. Zika virus was transmitted by sexual contact in Texas, health officials report. BMJ. 2016;352: i720 10.1136/bmj.i720 26848011

[13] Boyer S, Calvez E, Chouin-Carneiro T, Diallo D, Failloux AB. An overview of mosquito vectors of Zika virus. Microbes Infect. 2018;20: 646–660. 10.1016/j.micinf.2018.01.006 29481868

[14] Epelboin Y, Talaga S, Epelboin L, Dusfour I. Zika virus: an updated review of competent or naturally infected mosquitoes. PLoS Negl Trop Dis. 2017;11: e0005933 10.1371/journal.pntd.0005933 29145400 PMC5690600

[15] Akoua-Koffi C, Diarrassouba S, Bénié VB, Ngbichi JM, Bozoua T, Bosson A, et al Investigation surrounding a fatal case of yellow fever in Côte d’Ivoire in 1999. Bull Soc Pathol Exot. 2001;94: 227–230. 11681215

[16] Diallo D, Sall AA, Diagne CT, Faye O, Faye O, Ba Y, et al Zika virus emergence in mosquitoes in Southeastern Senegal, 2011. PLoS One. 2014;9: e109442 10.1371/journal.pone.0109442 25310102 PMC4195678

[17] Ferreira-de-brito A, Ribeiro IP, Moraes De Miranda R, Surubi Fernandes R, Silva Campos S, Antunes Barbosa da Silva K, et al First detection of natural infection of *Aedes aegypti* with Zika virus in Brazil and throughout South America. Mem Inst Oswaldo Cruz. 2016;111: 655–658. 10.1590/0074-02760160332 27706382 PMC5066335

[18] Guerbois M, Fernandez-Salas I, Azar SR, Danis-Lozano R, Alpuche-Aranda CM, Leal G, et al Outbreak of Zika Virus Infection, Chiapas State, Mexico, 2015, and First Confirmed Transmission by *Aedes aegypti* Mosquitoes in the Americas. J Infect Dis. 2016;214: 1349–1356. 10.1093/infdis/jiw302 27436433 PMC5079363

[19] da Costa CF, da Silva AV, do Nascimento VA, de Souza VC, Monteiro DC da S, Terrazas WCM, et al Evidence of vertical transmission of Zika virus in field-collected eggs of *Aedes aegypti* in the Brazilian Amazon. PLoS Negl Trop Dis. 2018;12: 1–12. 10.1371/journal.pntd.0006594 30011278 PMC6075777

[20] Cevallos V, Ponce P, Waggoner JJ, Pinsky BA, Coloma J, Quiroga C, et al Zika and Chikungunya virus detection in naturally infected *Aedes aegypti* in Ecuador. Mem Inst Oswaldo Cruz. 2018;177: 74–80. 10.1016/j.actatropica.2017.09.029 28982578

[21] Weger-Lucarelli J, Rückert C, Chotiwan N, Nguyen C, Garcia Luna SM, Fauver JR, et al Vector competence of American mosquitoes for three strains of Zika virus. PLoS Negl Trop Dis. 2016;10: 1–16. 10.1371/journal.pntd.0005101 27783679 PMC5081193

[22] Chouin-Carneiro T, Vega-Rua A, Vazeille M, Yebakima A, Girod R, Goindin D, et al Differential susceptibilities of *Aedes aegypti* and *Aedes albopictus* from the Americas to Zika virus. PLoS Negl Trop Dis. 2016;10: 1–11. 10.1371/journal.pntd.0004543 26938868 PMC4777396

[23] Ciota AT, Bialosuknia SM, Zink SD, Brecher M, Ehrbar DJ, Morrissette MN, et al Effects of Zika virus strain and *Aedes* mosquito species on vector competence. Emerg Infect Dis. 2017;23: 1110–1117. 10.3201/eid2307.161633 28430564 PMC5512477

[24] Roundy CM, Azar SR, Rossi SL, Huang JH, Leal G, Yun R, et al Variation in *Aedes aegypti* mosquito competence for Zika virus transmission. Emerg Infect Dis. 2017;23: 625–632. 10.3201/eid2304.161484 28287375 PMC5367433

[25] Tellechea AL, Luppo V, Morales MA, Groisman B, Baricalla A, Fabbri C, et al Surveillance of microcephaly and selected brain anomalies in Argentina: Relationship with Zika virus and other congenital infections. Birth Defects Res. 2018;110: 1016–1026. 10.1002/bdr2.1347 29921033

[26] Dirección de Epidemiología. Boletín Integrado de Vigilancia N° 418 –SE 33 2018 [Internet]. C.A.B.A, Argentina; 2018. https://www.argentina.gob.ar/sites/default/files/biv_418_se33.pdf

[27] Ciota AT, Chin PA, Ehrbar DJ, Micieli MV, Fonseca DM, Kramer LD. Differential effects of temperature and mosquito genetics determine transmissibility of arboviruses by *Aedes aegypti* in Argentina. Am J Trop Med Hyg. 2018;99: 417–424. 10.4269/ajtmh.18-0097 29869610 PMC6090362

[28] Waggoner JJ, Gresh L, Vargas MJ, Ballesteros G, Tellez Y, Soda KJ, et al Viremia and clinical presentation in Nicaraguan patients infected with Zika virus, chikungunya virus, and dengue virus. Clin Infect Dis. 2016;63: 1584–1590. 10.1093/cid/ciw589 27578819 PMC5146717

[29] Goenaga S, Kenney JL, Duggal NK, Delorey M, Ebel GD, Zhang B, et al Potential for co-infection of a mosquito-specific flavivirus, Nhumirim virus, to block West Nile virus transmission in mosquitoes. Viruses. 2015;7: 5801–5812. 10.3390/v7112911 26569286 PMC4664984

[30] Medina F, Medina JF, Colon C, Vergne E, Santiago GA, Munoz-Jordan JL. Dengue virus: isolation, propagation, quantification, and storage. Curr Protoc Microbiol. 2012;27: 15D.2.1–15D.2.24. 10.1002/9780471729259.mc15d02s27 23184594

[31] R Core Team. R: a language and environment for statistical computing [Internet]. Vienna, Austria: R Foundation for Statistical Computing; 2018 https://www.r-project.org/

[32] Ott RL, Longnecker M. An introduction to statistical methods and data analysis. 6th ed Cengage Learning; 2010.

[33] Good PI. Resampling Methods. 3rd ed Birkhauser Basel; 2006.

[34] Wickham H, François R, Henry L, Müller K. Dplyr: A Grammar of Data Manipulation. R package version 0.7.6. 2018.

[35] Pan American Health Organization (PAHO), World Health Organization (WHO). Zika suspected and confirm cases reported by countries and territories in the Americas Cumulative cases, 2015–2017 [Internet]. Pan American Health Organization Washington, D.C; 2017 https://www.paho.org/hq/index.php?option=com_content&view=article&id=12390:zika-cumulative-cases&Itemid=42090&lang=en

[36] Smartt CT, Stenn TMS, Chen TY, Teixeira MG, Queiroz EP, Souza Dos Santos L, et al Evidence of zika virus RNA fragments in aedes albopictus (Diptera: Culicidae) field-collected eggs from Camaçari, Bahia, Brazil. J Med Entomol. 2017;54: 1085–1087. 10.1093/jme/tjx058 28419254

[37] Díaz-Quiñonez JA, López-Martínez I, Torres-Longoria B, Vázquez-Pichardo M, Cruz-Ramírez E, Ramírez-González JE, et al Evidence of the presence of the Zika virus in Mexico since early 2015. Virus Genes. 2016;52: 855–857. 10.1007/s11262-016-1384-0 27557815

[38] Di Luca M, Severini F, Toma L, Boccolini D, Romi R, Remoli ME, et al Experimental studies of susceptibility of Italian *Aedes albopictus* to Zika virus. Eurosurveillance. 2016;21: 30223 10.2807/1560-7917.ES.2016.21.18.30223 27171034

[39] Aliota MT, Peinado SA, Velez ID, Osorio JE. The wMel strain of Wolbachia Reduces Transmission of Zika virus by *Aedes aegypti*. Sci Rep. Nature Publishing Group; 2016;6: 28792 10.1038/srep28792 27364935 PMC4929456

[40] Fernandes RS, Campos SS, Ribeiro PS, Raphael LMS, Bonaldo MC, Lourenço-de-oliveira R. Culex quinquefasciatus from areas with the highest incidence of microcephaly associated with Zika virus infections in the Northeast Region of Brazil are refractory to the virus. Mem Inst Oswaldo Cruz. 2017;112: 577–579. 10.1590/0074-02760170145 28767975 PMC5530542

[41] Rückert C, Weger-Lucarelli J, Garcia-Luna SM, Young MC, Byas AD, Murrieta RA, et al Impact of simultaneous exposure to arboviruses on infection and transmission by *Aedes aegypti* mosquitoes. Nat Commun. 2017;8: 1–9. 28524874 10.1038/ncomms15412 PMC5454532

[42] Main BJ, Nicholson J, Winokur OC, Steiner C, Riemersma KK, Stuart J, et al Vector competence of *Aedes aegypti*, *Culex tarsalis*, and *Culex quinquefasciatus* from California for Zika virus. PLoS Negl Trop Dis. 2018;12: e0006524 10.1371/journal.pntd.0006524 29927940 PMC6013020

[43] Richards SL, Pesko K, Alto BW, Mores CN. Reduced infection in mosquitoes exposed to blood meals containing previously frozen flaviviruses. Virus Res. 2007;129: 224–227. 10.1016/j.virusres.2007.06.019 17686541 PMC2746366

[44] Micieli M V., Matacchiero AC, Muttis E, Fonseca DM, Aliota MT, Kramer LD. Vector competence of Argentine mosquitoes (Diptera: Culicidae) for West Nile virus (Flaviviridae: Flavivirus). J Med Entomol. 2013;50: 853–862. 10.1603/me12226 23926785 PMC3932752

[45] Gloria-Soria A, Ayala D, Bheecarry A, Calderon-Arguedas O, Chadee DD, Chiappero M, et al Global genetic diversity of *Aedes aegypti*. Mol Ecol. 2016;25: 5377–5395. 10.1111/mec.13866 27671732 PMC5123671

[46] Diagne CT, Diallo D, Faye O, Ba Y, Faye O, Gaye A, et al Potential of selected Senegalese *Aedes spp*. mosquitoes (Diptera: Culicidae) to transmit Zika virus. BMC Infect Dis. BMC Infectious Diseases; 2015;15: 492 10.1186/s12879-015-1231-2 26527535 PMC4629289

[47] Dirección de Epidemiología. Boletín Integrado de Vigilancia N° 375 –SE 35 2017 [Internet]. C.A.B.A, Argentina; 2017. http://www.msal.gob.ar/images/stories/boletines/boletin_integrado_Vigilancia_375.pdf

[48] Lourenço-de-oliveira R, Rua AV, Vezzani D, Willat G, Vazeille M, Mousson L, et al *Aedes aegypti* from temperate regions of South America are highly competent to transmit dengue virus. BMC. 2013;13: 610 10.1186/1471-2334-13-610 24373423 PMC3929315

[49] Dirección de Epidemiología. Boletín Integrado de Vigilancia N° 320 –SE 30 2016 [Internet]. C.A.B.A, Argentina; 2016. https://www.argentina.gob.ar/sites/default/files/boletin-integrado-de-vigilancia-n320-se30.pdf

[50] Fischer S, De Majo MS, Quiroga L, Paez M, Schweigmann N. Long-term spatio-temporal dynamics of the mosquito Aedes aegypti in temperate Argentina. Bull Entomol Res. 2017;107: 225–233. 10.1017/S0007485316000869 27876100

[51] Vezzani D, Carbajo AE. *Aedes aegypti*, *Aedes albopictus*, and dengue in Argentina: current knowledge and future directions. Mem Inst Oswaldo Cruz. 2008;103: 66–74. 10.1590/S0074-02762008005000003 18327504

[52] Chuchuy A, Rodriguero MS, Ferrari W, Ciota AT, Kramer LD, Micieli M V. Biological characterization of *Aedes albopictus* (Diptera: Culicidae) in Argentina: implications for arbovirus transmission. Sci Rep. 2018;8: 1–8. 29568046 10.1038/s41598-018-23401-7 PMC5864732

[53] Richards SL, Anderson SL, Lord CC, Smartt CT, Tabachnick WJ. Relationships between infection, dissemination, and transmission of West Nile virus RNA in *Culex pipiens quinquefasciatus* (Diptera: Culicidae). J Med Entomol. 2012;49: 132–42. 10.1603/me10280 22308781 PMC3320798

[54] Rossi GC. Annotated checklist, distribution, and taxonomic bibliography of the mosquitoes (Insecta: Diptera: Culicidae) of Argentina. Check List. 2015;11: 1712 10.15560/11.4.1712

[55] Holland J, Domingo E. Origin and evolution of viruses Virus Genes. Boston: Kluwer Academic Publishers; 1998 pp. 13–21. http://link.springer.com/10.1023/A:1007989407305%5Cnpapers3://publication/doi/10.1023/A:1007989407305 10.1023/a:1007989407305 9562888

[56] Weaver SC, Reisen WK. Present and future arboviral threats. Antivir Res. 2010;85: 328 10.1016/j.antiviral.2009.10.008 19857523 PMC2815176

[57] De Lamballerie X, Leroy E, Charrel RN, Ttsetsarkin K, Higgs S, Gould EA. Chikungunya virus adapts to tiger mosquito via evolutionary convergence: a sign of things to come? Virol J. 2008;5: 33 10.1186/1743-422X-5-33 18304328 PMC2266737

[58] Zou G, Puig-Basagoiti F, Zhang B, Qing M, Chen L, Pankiewicz KW, et al A single-amino acid substitution in West Nile virus 2K peptide between NS4A and NS4B confers resistance to lycorine, a flavivirus inhibitor. Virology. Elsevier Inc.; 2009;384: 242–252. 10.1016/j.virol.2008.11.003 19062063 PMC5388927

[59] McMullen AR, Albayrak H, May FJ, Davis CT, Beasley DWC, Barrett ADT. Molecular evolution of lineage 2 West Nile virus. J Gen Virol. 2013;94: 318–325. 10.1099/vir.0.046888-0 23136360 PMC3709619

